# Brains in Competition: Improved Cognitive Performance and Inter-Brain Coupling by Hyperscanning Paradigm with Functional Near-Infrared Spectroscopy

**DOI:** 10.3389/fnbeh.2017.00163

**Published:** 2017-08-31

**Authors:** Michela Balconi, Maria E. Vanutelli

**Affiliations:** Research Unit in Affective and Social Neuroscience, Department of Psychology, Catholic University of Milan Milan, Italy

**Keywords:** hyperscanning, competition, emotion, cognition, fNIRS

## Abstract

Hyperscanning brain paradigm was applied to competitive task for couples of subjects. Functional Near-Infrared Spectroscopy (fNIRS) and cognitive performance were considered to test inter-brain and cognitive strategy similarities between subjects (14 couples) during a joint-action. We supposed increased brain-to-brain coupling and improved cognitive outcomes due to joint-action and the competition. As supposed, the direct interaction between the subjects and the observed external feedback of their performance (an experimentally induced fictitious feedback) affected the cognitive performance with decreased Error Rates (ERs), and Response Times (RTs). In addition, fNIRS measure (oxyhemoglobin, O2Hb) revealed an increased brain activity in the prefrontal cortex (PFC) in post-feedback more than pre-feedback condition. Moreover, a higher inter-brain similarity was found for the couples during the task, with higher matched brain response in post-feedback condition than pre-feedback. Finally, a significant increased prefrontal brain lateralization effect was observed for the right hemisphere. Indeed the right PFC was more responsive with similar modalities within the couple during the post-feedback condition. The joined-task and competitive context was adduced to explain these cognitive performance improving, synergic brain responsiveness within the couples and lateralization effects (negative emotions).

## Introduction

Competition essentially implies a social dynamic that requires a comparison between two or more subjects during an interpersonal performance. Previous research suggested a crucial role of competitive social interactions in achieving accurate self-representation of our social position. Conversely, it was found that social perception affects performance during situations that require to compare our own behavior with that of others (Munafò et al., 2005). That is, the analysis of our social role in competition may influence the cognitive performance by improving or decreasing the actual outcomes (Munafò et al., 2005).

About the brain contribution, it was observed that an extended neural network, including limbic areas, the prefrontal cortex (PFC) and striatal structures, may represent the behavioral cognitive and emotional and correlates of social interactions, respectively (Levitan et al., 2000). Preliminary evidence in support of this neural mechanism of the social brain comes from previous studies exploring the structures and functions of brain areas associated with social representation, social ranking and self-efficacy. Specifically, both dorsal (DLPFC) and ventral (VLPFC) portions of the lateral PFC are generally involved in response to social status inference and interpersonal tasks (Chiao et al., 2009b; Balconi and Pagani, 2014, 2015). The activation of DLPFC and VLPFC during social interactions probably represents the recruitment of brain areas that apply top-down control over some processes, such as emotional behavior in response to social demands (Marsh et al., 2009; Balconi and Vanutelli, 2016). Indeed these brain areas are generally associated with socio-emotional regulation and behavioral inhibitory mechanisms.

Recent research on cooperation/competition also showed enhanced cortico-cortical communication and interconnections between these prefrontal areas. For example, the effects of competitive tasks in more than one brain was recently explored (Decety et al., 2004; Liu et al., 2015; Cui et al., 2016). In addition, some studies confirmed that the social context jointly affects subjects’ reactions to their environment and consequently their brain activity. For example it was noted that one’s own action planning is facilitated during cooperation since others’ actions are joined with our actions, in opposition to competitive conditions (Knoblich and Jordan, 2003; Sebanz et al., 2003).

Therefore the cortical activity is also modulated based on the different forms of social interaction, since competitive or cooperative situations are qualitatively distinct contexts. Indeed, it should be noted that cooperation and competition are two basic types of interpersonal interaction (Decety et al., 2004). That is, based on the interactive condition (cooperation vs. competition) people may either facilitate or hinder the goals of others. Specifically competition, as a social-evaluative phenomenon, can increase the amount of cognitive resources beyond what is needed to simply execute the task demands. In particular, the cognitive effort could be increased in competition when subjects have potentially contrasting goals (De Cremer and Stouten, 2003; Decety et al., 2004). We previously focused on cooperation with specific measures (EEG and neuroimaging near-infrared spectroscopy (NIRS); Balconi and Vanutelli, 2017a,b) and then we applied EEG and Functional NIRS (fNIRS) to study competition.

It was also found that competition may improve the effective cognitive performance and the self-perception of higher social position (Goldman et al., 1977). The higher demand may explain how competition affects performance (with an “improving effect”) and brain responsiveness due to the attendant modifications of the subject’s mental condition and underlying neural activities (Rietschel et al., 2011). More specifically, the self-perception during competition may affect the subjective internal judgment and manifest as an increased cerebral responsiveness in those areas related to competitive conditions, and positively affect the performance outcomes.

However, whereas the available previous results indicate that social exchanges involve a specific network of cortical areas, further analysis is required to clarify the specific contribution of the brain structures in different social conditions, i.e., when subjects compete toward a personal goal during a joint action. Second, the presence of a real interlocutor may affect the inter-brain responsiveness and cognitive outcomes, as suggested by hyperscanning research (Konvalinka and Roepstorff, 2012). In a recent study Cui et al. (2016) have measured the prefrontal activation during cooperative and competitive tasks by using NIRS. Dyads of participants were asked to press two keys either simultaneously (to obtain synchronized action in cooperative condition), or as fast as possible to obtain a better result than their partner during competitive condition. The participants showed increased inter-brain synchronization in the right superior frontal areas during cooperation, but not competition: such result emerged because of the necessity to model others’ behavior during a cooperative task. It should also be considered that the increase in cortico-cortical communication was high and significant, and involved heightened responses between all non-motor areas with strategy planning regions (such as prefrontal areas).

Third, it should be considered whether and how an increase in brain activity and cognitive performance is specifically promoted by an external feedback which is able to manipulate the cognitive performance though self-other evaluation. In fact no previous research has considered the social environment and the cognitive outcomes by using a direct competitive task. Generally, previous studies implied only single subjects and their isolated performance in abstract social tasks, since they did not include paired joint actions and interactive tasks. In other cases research explored the response in asynchronous conditions (subsequential response by the participants) by two or more subjects interacting each other (Boone et al., 1999; Decety et al., 2004). In this regard, the hyperscanning approach introduces an innovative perspective to explore two interacting brains (Holper et al., 2012; Konvalinka and Roepstorff, 2012). However, when an hyperscanning paradigm was used, it was applied only in response to cognitive performance without a specific interactive feedback (Saito et al., 2010; Dommer et al., 2012; Cui et al., 2016).

Therefore, to summarize, compared to previous research, two relevant aspects were underestimated and deserve to be considered to evaluate inter-subjective brain activity and the cognitive performance during competition: the presence of a dual interaction, and the feedback furnished by the social context to (fictitiously) represent the effectiveness of the joint action. That is, the effect of an external feedback (positive or negative feedback about the competitive performance) on the inter-brain responses and cognitive performance was not adequately considered. An external feedback is supposed to modify the self-representation, the effective cognitive outcomes and the brain responsiveness to social contexts (Montague et al., 2002). In the present study the performance was manipulated in a vis-à-vis competitive situation which stressed the subjects’ ability to win and to perform better than the partner. Compared to other studies (Zink et al., 2008), we planned a more ecological and realistic scenario where subjects were asked to directly compare their outcomes with the other partner by monitoring their performance. Specifically this request underlined the necessity to increase subject’s effectiveness during the task (“your performance is better than…”). In this regard we formulated a clear and reinforced social condition based on cognitive skill during a dyadic interaction. Second, brain-to-brain coupling effect induced by the competitive task has to be explored, by using an adequate hyperscanning paradigm, which is able to reveal the common strategies applied by the participants to obtain a better performance and the effects of this synergic planning. To test these double effects, fNIRS was applied to acquire subjects’ brain response during a task performed simultaneously in paired subjects. Classical neuroimaging approach (i.e., functional Magnetic Resonance, fMRI) was not able to exhaustively show the social nature of the inter-personal processes since the temporal course of such activation was scarcely addressed. fNIRS measure has a resolution which is considered high enough for monitoring event-related fNIRS responses (Elwell et al., 1993; Montague et al., 2002; Decety et al., 2004; Dötsch and Schubö, 2015). More importantly, fNIRS proved to be much more suitable for ecological hyperscanning applications since it imposes significantly milder physical burdens than other techniques such as fMRI, it is not noisy or uncomfortable, and is robust to exogenous noise thus permitting interactive contexts (Balconi and Molteni, 2015).

Therefore, based on our hypotheses, the artificially increased performance during competition may effectively modulate the behavioral performance in social contexts, with improved outcomes mainly after receiving the feedback. Therefore, a consistent better performance should be found for the post-feedback condition (that is in the case of perception of improved outcomes), as a result of a higher reinforcing situation. Second, the cortical effect of these social and cognitive representation processes are hypothesized to be supported by the PFC (Hall et al., 2005; Chiao et al., 2009a; Balconi and Pagani, 2014, 2015), with significant higher responsiveness of the PFC mainly after the feedback. Third, we intended to study inter-brain activity in competitive conditions and in relationship with the positive (improved) feedback. Indeed we expected a higher brain-to-brain coupling induced by the feedback, which may induce a more synergic activity between the subjects.

## Materials and Methods

### Subjects

Fourteen couples of subjects (28 subjects, all undergraduate students: *M* = 23.78, SD = 1.98 years old) were recruited for the present research. Each couple was composed by two players of the same gender matched for age. The subjects were all right-handed, with normal or corrected-to-normal visual acuity. To exclude history of psychopathology Beck Depression Inventory (BDI-II, Beck et al., 1996) was administered to the participants or immediate family. Moreover State-Trait-Anxiety-Inventory (STAI, Spielberger et al., 1970) was submitted in the post-experimental session. No neurological or psychiatric pathologies were revealed. The research was approved by the local ethics committee of the Department of Psychology, Catholic University of Milan. The subjects gave informed written consent to participate in the study in accordance with the 1964 Helsinki Declaration and its later amendments or comparable ethical standards. No payment was provided for subjects’ performance.

### Procedure

Subjects were seated with monitor in front of them (positioned approximately 60 cm) in a moderately darkened room. Participants were seated side-by-side and separated by a black screen in order to not seeing each other. They performed a cognitive task for sustained selective attention (modified version of Balconi and Pagani, 2014).

### Task

**Figure 1:**
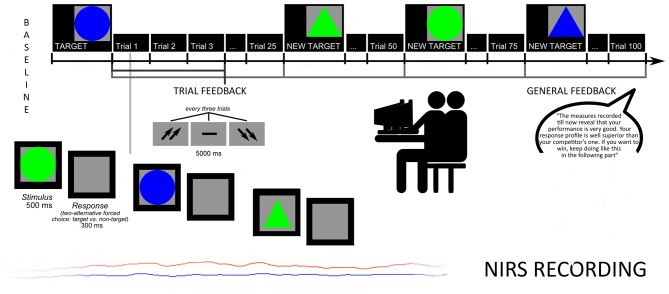
Experimental procedure: task structure and oxyhemoglobin (O2Hb) measures.

Subjects were taught that specific attentional measures were registered to evaluate the subjective skills. They were required to recognize target stimuli from non-targets, based on four different combinations of shape and color: circles or triangles, green or blue. The target remained on the video until subjects were able to memorize it. Then, stimuli were displayed one after another. Size and color features changed every experimental block, each composed by 25 trials. Subjects were asked to press a left/right button after each stimulus to decide for target/non target. Each stimulus was displayed on the screen for 500 ms (300 ms inter-stimulus interval, ISI), and each trial was constituted by three stimuli. After each trial a feedback appeared on the screen in the form of two up-arrows (better performance than the competitor); a dash (comparable performance); or two down-arrows (worse performance). The feedback lasted 5000 ms, followed by an inter-trial interval (ITI) of 5000 ms. The task was subdivided in two sub-sessions: the first without a specific feedback to subject’s performance (four blocks of stimuli before the feedback, for a total of 100 trials); the second preceded by the feedback about the performance (four blocks of stimuli with the feedback, for a total of 100 trials; Figure 1).

To increase subjects’ intrinsic motivation, they were told that accuracy, number of errors (Error Rate, ER) and response times (RTs) were usually used to screen future professional career success in term of teamwork abilities. Moreover, participants were told that the final cognitive outcome was based on the ability to produce a better performance than the competitor, in order to highly stress the competitive nature of the task.

Halfway, participants received also a synthetic feedback of their cognitive performance. Both feedbacks (both trial-related and general feedback) were prearranged (without the awareness of the participants), and participants were told that their outcome was “well above (score with 91% in terms of speed, and 92% in terms of accuracy)”. In addition they were pressed to maintain their higher performance level during the course of the task (“*The measures recorded till now reveal that* *your performance is very good. Your response profile is well superior to your competitor’s one. If you want to win, keep going like this in the following part*”). Trial feedbacks constantly reinforced them about their high and competitive performance by presenting the up-arrows (70% of the cases) and the dash or the down-arrows (30% of the cases, and they were mainly positioned at the beginning of the task), to make the outcome more credible and plausible.

Finally, after each block, subjects were required to rank their outcome and perceived self-efficacy on a 7-point Likert scale (1 = most decreased performance; 7 = most improved performance). As reported in a post-experimental phase, the participants were strongly engaged in the social and ranking process. Participants were also requested to self-report their degree of trust based on the external feedback. They showed very high trust (92%) and a self-represented relevance of the task for their social position (94%).

#### Performance Scoring

The RTs (ms) measures were registered from the stimulus onset, and ERs were calculated as the total number of incorrect detections out of the total trial, for each experimental category (therefore higher values represented higher number of incorrect responses).

#### fNIRS

**Figure 2:**
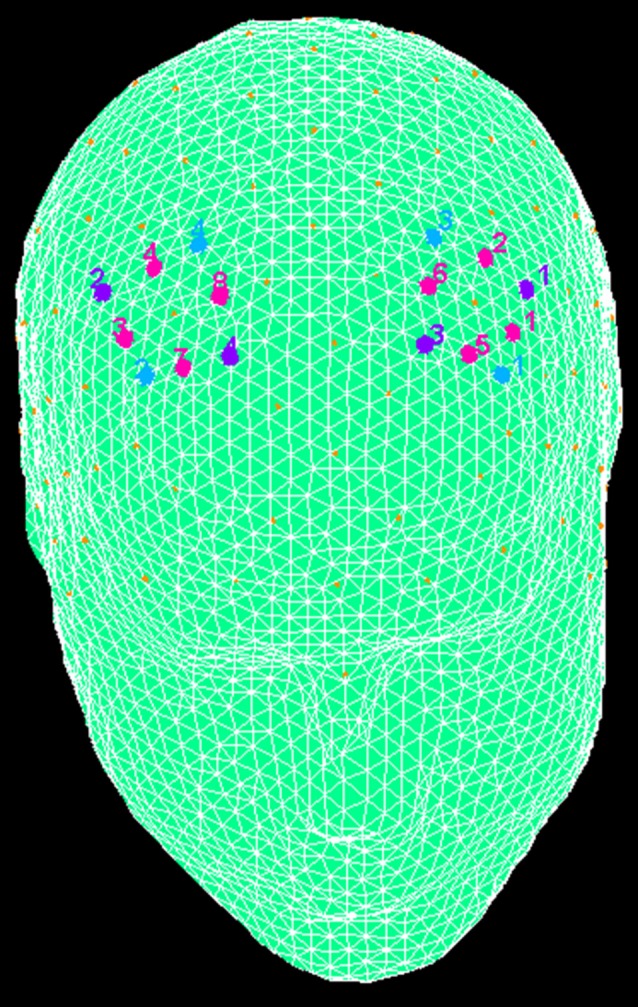
The location of near-infrared spectroscopy (NIRS) channels. The emitters were placed on positions FC3-FC4 (purple, number 1 and 2) and F1-F2 (number 3 and 4), while detectors were placed on FC1-FC2 (blue, number 3 and 4) and F3-F4 (number 1 and 2). Resulting channels are displayed in pink color.

fNIRS recordings were conducted with NIRScout System (NIRx Medical Technologies, LLC. Los Angeles, CA, USA) with an 8-channel array of optodes placed on the prefrontal area (four light sources/emitters and four detectors). Emitters were placed over FC3-FC4 and F1-F2 positions, while detectors were placed on FC1-FC2 and F3-F4 positions (Figure 2). Emitter-detector distance was maintained at 30 mm for contiguous optodes and near-infrared light of two wavelengths (760 and 850 nm) were considered. According to the international 10/5 system, NIRS optodes were placed on the subject’s head using a NIRS-EEG compatible cup. Changes in the concentration of oxygenated (O2Hb) and deoxygenated hemoglobin (HHb) from a 120 s resting baseline were acquired. By using NIRStar Acquisition Software, Signals obtained from the eight NIRS channels were acquired (sampling rate of 6.25 Hz), then transformed in values for the changes in the concentration of oxygenated and deoxygenated HHb in each channel (scaled in mmol*mm).

The raw O2Hb and HHb data from each channel were digitally band-pass filtered at 0.01–0.3 Hz. Then, the mean concentration of each channel was calculated (from the trial onset for the following 5 s) as the average across trials. According to the mean concentrations in the time series, the effect size was calculated for each channel and participant in every experimental condition. The Cohen’s z effect sizes were obtained as the difference of the means of the baseline and trial divided by the standard deviation (SD) of the baseline, as reported in the formula: *d* = (m1 − m2)/s. m1 and m2 were the mean concentration values during baseline and trial, respectively, and s the SD of the baseline. The baseline was calculated considering the 5 s period immediately before the trial beginning. Then, in order to increase the signal-to-noise ratio, the effect sizes obtained from the eight channels were averaged. Although NIRS raw data were originally relative values and for his reason they could not be directly averaged across experimental conditions (subjects or channels), effect sizes normalized data could be averaged regardless of the unit since the effect size is not affected by differential pathlength factor (DPF; Schroeter et al., 2003; Matsuda and Hiraki, 2006; Shimada and Hiraki, 2006).

### Data Analysis

Three levels of analyses were performed for behavioral (ER; RTs) and neurophysiological (fNIRS, O2Hb measures) measures.

For the first level of analysis, a repeated measure ANOVA with one factor (Condition, Cond: pre vs. post feedback) was applied to ERs and RTs data. A second ANOVA was applied to O2Hb dependent measure, with repeated factors Cond and Lateralization (Lat: left vs. right). This preliminary set of ANOVAs was finalized to test the general effect of Condition (for ERs and RTs) and Condition and Lateralization (for O2Hb) in the whole sample.

For the second level, a similarity measure for continuous data was applied to each couple of subjects in pre- and post-feedback condition (for the 100 trials). These similarity measures for interval data, i.e., Pearson correlation as a measure of distance between vectors, finds the ratio between the covariance and the SD of both subjects (Sheldon, 2014). By using this measure specific similarities between each couple of subjects was monitored for cognitive (ERs and RTs) and O2Hb dependent measures.

For the third level, to analyze the systematic effect of the independent within subjects factors Cond on the similarities coefficients, repeated measure ANOVAs were applied to the coefficients calculated for ERs and RTs as dependent variables. In addition to Cond, Lateralization factor was added in the case of O2Hb coefficients as dependent measure.

For all the ANOVA, the Greenhouse–Geisser epsilon was used for degrees of freedom correction where appropriate. *Post hoc* comparisons (contrast analyses) were used when necessary and Bonferroni test was used in the case of multiple comparisons.

To exclude a possible learning effect due to pre-/post feedback condition, a preliminary analysis was conducted, comparing distinctly the first groups of intervals (four pre-feedback intervals, for each 25 trials) and the second group of intervals (four post feedback intervals, for each 25 trials) for all the dependent variables (RTs, ERs, O2Hb). Since no significant differences among the four intervals, respectively for before and after feedback condition, were found, we did not include this factor in the successive phases of the analysis.

## Results

### ANOVA (Raw Data)

#### ER and RTs

**Figure 3:**
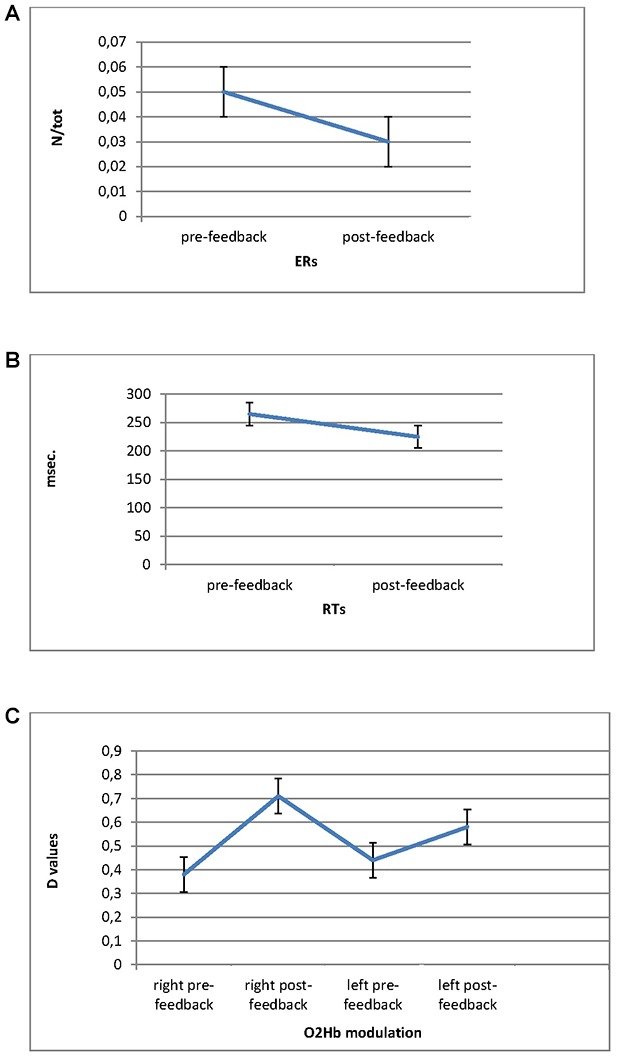
Error Rates (ERs; **A**) and Response Times (RTs; **B**) modulation as a function of pre-feedback and post-feedback, with decreased values in post-feedback. **(C)** O2Hb modulation (D values) for Condition and Lateralization. Post-feedback condition revealed increased D values for the right prefrontal cortex (PFC).

For ER measure, ANOVA indicated significant effect for Cond (*F*_(1,27)_ = 8.90, *p* ≤ 0.001, *η*^2^ = 0.37). Indeed ER decreased in post-feedback (*M* = 0.03; SD = 0.009) compared to pre-feedback (*M* = 0.05; SD = 0.01; Figure 3A).

For RTs, ANOVA revealed significant main effect for Cond (*F*_(1,27)_ = 9.05, *p* ≤ 0.001, *η*^2^ = 0.39), showing reduced RTs for post-feedback (*M* = 223; SD = 0.25) compared to pre-feedback (*M* = 258; SD = 0.31; Figure 3B).

#### O2Hb

The successive ANOVAs were applied to d measure for both O2Hb and HHb-values. Since the analysis on HHb did not show any significant results only statistical results for O2Hb were reported. The data over left (Ch1: FC3-F3; Ch2: FC3-FC1; Ch5: F1-F3; Ch6: F1-FC1) and right (Ch3: FC4-F4; Ch4: FC4-FC2; Ch7: F2-F4; Ch8: F2-FC2) channels were averaged.

ANOVA showed Lat × Cond significant interaction effect (*F*_(1,27)_ = 11.32, *p* ≤ 0.001, *η*^2^ = 0.40) with increased right brain responsiveness for post-feedback (*M* = 0.72; SD = 0.02) compared to pre-feedback condition (*M* = 0.38; SD = 0.01; Figure 3C).

### Similarity Measures

#### ER and RTs

**Figure 4:**
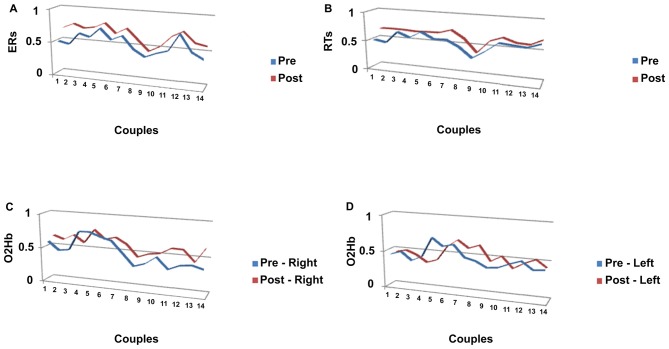
Similarity measures (Pearson coefficients) for couples of subjects for **(A)** ERs; **(B)** RTs, a function of pre-feedback and post-feedback; **(C)** O2Hb variable as a function of right **(C)** and left **(D)** hemisphere.

The Pearson similarity coefficients (Fisher’s z transform) were reported in the following Figures 4A–B for each couple of subjects in pre- and post-feedback. As indicated in Figure 4A, for ER, five couples showed significant coefficients for the pre-feedback condition, whereas 10 couples showed significant coefficients for the post-feedback condition. Figure 4B indicates the coefficients for RTs measures. As reported, nine couples revealed significant joined RTs modulation for the pre-feedback condition, whereas 13 couples showed significant coefficients in post-feedback.

#### O2Hb

Significant Pearson coefficients were found in pre-feedback condition: six couples in the right and four in the left hemisphere revealed significant coefficients, whereas 12 couples were matched in post-feedback in the right side and five couples in the left side (Figures 4C,D).

### ANOVA on Similarity Measures

The third level of analysis considered the Pearson coefficients derived for ER, RTs and O2Hb as dependent measure in the repeated measures ANOVAs.

#### ER and RTs Coefficients

**Figure 5:**
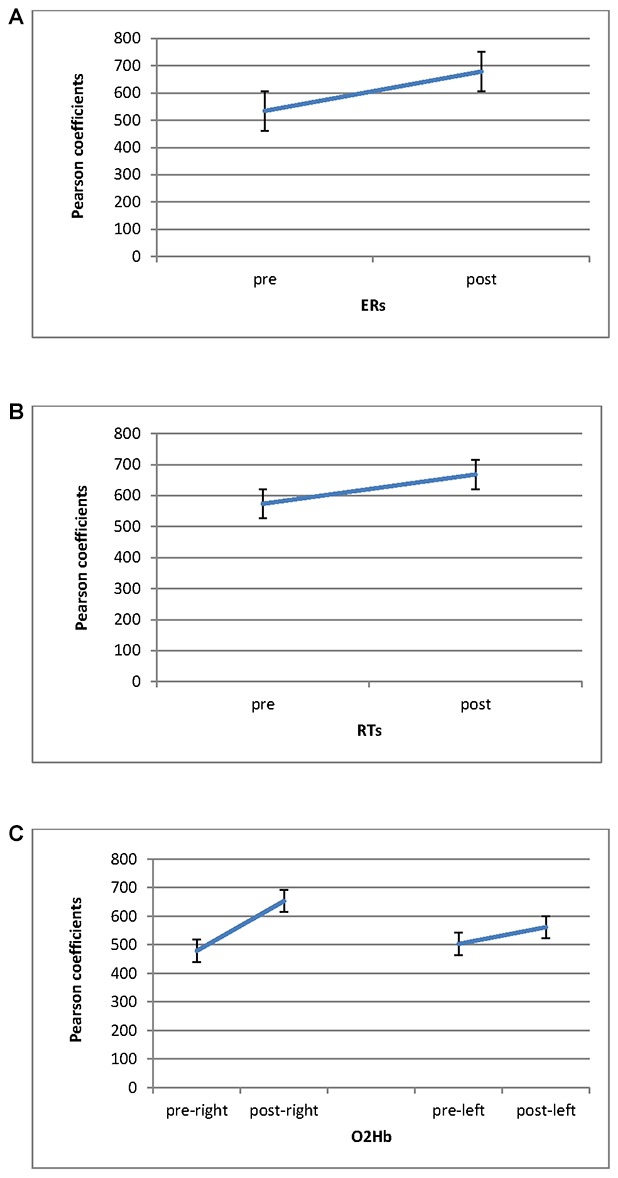
Pearson coefficients as a function of pre-feedback and post-feedback for **(A)** ERs; **(B)** RTs measures, with decreased ERs and RTs for post-feedback; **(C)** O2Hb. It was observed an increasing of O2Hb for post-feedback in the right hemisphere.

Significant differences in ER were found for Cond (*F*_(1,27)_ = 9.06, *p* ≤ 0.001, *η*^2^ = 0.39), with increased Pearson coefficients values in post-feedback (*M* = 0.68; SD = 0.01) than pre-feedback (*M* = 0.54; SD = 0.01) condition (Figure 5A). For RTs, a significant result was found for Cond (*F*_(1,27)_ = 7.76, *p* ≤ 0.001, *η*^2^ = 0.34), with increased Pearson values in post-feedback (*M* = 0.67; SD = 0.03) than pre-feedback (*M* = 0.57; SD = 0.02) condition (Figure 5B).

#### O2Hb Coefficients

Significant effect was found for Cond (*F*_(1,27)_ = 8.79, *p* ≤ 0.001, *η*^2^ = 0.36) and Cond × Lat (*F*_(1,27)_ = 7.52, *p* ≤ 0.001, *η*^2^ = 0.34). Indeed, increased coefficients were revealed in post-feedback (*M* = 0.60; SD = 0.02) than pre-feedback condition (*M* = 0.49; SD = 0.01). Second, about the interaction effect, during post-feedback the right hemisphere showed higher coefficient values (*M* = 0.67; SD = 0.02) compared to the left hemisphere (*M* = 0.57; SD = 0.01; *F*_(1,27)_ = 7.12, *p* ≤ 0.001, *η*^2^ = 0.34). In addition the right hemisphere registered increased Pearson values in post-feedback (*M* = 0.67; SD = 0.01) than in pre-feedback (*M* = 0.47; SD = 0.03; *F*_(1,27)_ = 7.43, *p* ≤ 0.001, *η*^2^ = 0.35; Figure 5C).

## General Discussion

The present research explored the effects of a competitive joint-action on cognitive performance and brain activity by using a hyperscanning paradigm. Specifically, inter-brain similarities measures were acquired in couples of subjects during a competitive task, by using fNIRS. Based on our results, the following effects were observed. A first main effect was the systematic prefrontal (PFC) increased activity when a positive reinforce (post-feedback) was furnished to the participants about their performance. Indeed significant increased PFC activity in response to a positively reinforced joint action was found for all participants when compared to pre-feedback condition. Second, a better performance for both RTs and ER measures was revealed after the reinforcing feedback. Third, a higher inter-brain similarity was found for the couples after the feedback. Specifically when participants perceived (experimentally induced) to have performed better, a homologous and similar brain response was produced, with higher coherent PFC activity within the couple. Finally, it should be noted that a significant prefrontal brain lateralization effect was present, with the right hemisphere being more engaged in post-feedback condition.

About the first result, previous evidence revealed that prefrontal areas are crucial in social status monitoring and joint actions (Karafin et al., 2004; Haruno and Kawato, 2009; Suzuki et al., 2011). Also, using EEG-based hyperscanning technique, specific DLPFC activation emerged during reciprocal interaction in iterated Prisoner’s Dilemma paradigm (De Vico Fallani et al., 2010). In the present research we observed a similar effect, with significant increased PFC activity in response to positively reinforced joint action during the cognitive task. This prefrontal brain area was hypothesized to have an evolutionary role in social perception mainly when hierarchy in social groups is crucial (Chiao et al., 2009a). Therefore we may suggest that this area has dedicated mechanisms to perceive social position and interaction significance during an interpersonal task.

More interestingly, the post-feedback condition induced an increased PFC responsiveness than pre-feedback condition. In fact, we observed that the PFC was mainly implicated when subjects were informed on their efficient interaction. This fact may indicate a central role of this prefrontal area in the case of a positive self-perception (to be a good performer) within a social situation where the competition is relevant and stressed. It is interesting to note that this “improved brain effect” was also accompanied by a significant increased cognitive performance (decreased ER and RTs). Indeed it was found that subjects highly improved their cognitive outcomes in response to the external reinforce. Due to these results we may suppose that the improved self-representation in term of social ranking and social position may have enhanced the real subjective performance. It should also be noted that the cortical and behavioral data showed to be matched, with a similar trend of higher activity for both behavior and cortical activity, which underlined the main effect of the (artificially) induced positive social reinforce on the inter-subjective joint performance.

About the inter-brain relation, we observed a consistent and relevant increased brain-to-brain coupling for the dyads, mainly in concomitance with the positive social feedback. That is, this homologous inter-brain activity emerged in post-feedback condition for most of the couples. Therefore we may state that the externally induced reinforcing condition influenced the joint cortical responsiveness. That is, it could be suggested that, although the task was competitive, the self-perceived efficacy produced a sort of “glue” between the two brains, orienting the subjects on the same direction. Therefore, the present results provides initial evidence for the hypothesis of a significant inter-brain effect during competitive tasks and offer suggestions for future studies examining the extent to which the competition in two brains is selectively related to a better cognitive joint performance for the two inter-agents.

This fact was further underlined by the significant effect of positive feedback on the cognitive joint-performance. Indeed the common strategy was evident also for the cognitive measures (RTs and ER) in addition to the brain measures. Higher similarity coefficients were found for the cognitive variables, thus underlining the impact of the external feedback on both the hemodynamic and cognitive level. In other terms, we may suppose that the external reinforce may have modulated the effective joint-behavior inside the couple, with relevant convergence of the increased performance by the two inter-agents.

About inter-subjective joint neural activity it should be noted that this prominent effect was mainly observed for the right hemisphere with respect to the left one. This result may be understood taking into account the social role of PFC and the lateralized effect observed in previous studies (Balconi et al., 2012). At this regard, we may consider the increased responsiveness in the right hemisphere as a possible marker of the competitive goal, oriented toward the maximization of the personal profit. Indeed, as previously demonstrated, the prefrontal asymmetry in favor of the right hemisphere may represent the withdrawal motivation in opposition to approach motivation (Davidson, 1993; Jackson et al., 2003; Urry et al., 2004; Balconi and Mazza, 2010; Harmon-Jones et al., 2010; Koslow et al., 2013).

An alternative second explanation of the present result may relate the increased right hemisphere responsiveness to a significant increasing of more negative and avoidance emotions toward the competitor, linked to the competitive condition. As previously shown, the right hemisphere is supporting the aversive situations where the subjects are required to manage the conflictual and potential divergent goals (Balconi et al., 2012). Therefore, a sort of a “negative echo” may be induced by the individualistic and competitive aims of the task for each subject, with a significant increasing of more withdrawal attitudes. Consequently, prefrontal brain activity can be regarded to be highly involved in the processing of emotional behavior which affects the competitive context (Adolphs, 2002; Chiao et al., 2009a). However actually few studies have tried to study the emotional effects of competition on brain activity, taking into consideration the role of emotions on the cortical response (and on inter-brains responsiveness) when it responds to social situations as competition of cooperation. For this reason future research should better explore the distinct effect of emotions and competition on the cortical responsiveness to disambiguate their reciprocal relation.

However some limitations could be suggested for the present study. First, some adjunctive analyses could be used, to elucidate the gender effect in interactions, since the couples were composed by males or females. Second, a more accurate analysis for the dynamical changes of the inter-subjective strategy during the task should be conducted, in order to verify the progression of the learning mechanisms related to the inter-brain and cognitive processes. Finally, future research should better explore the effect of competitive in comparison with cooperative task, to verify the significant differences in brain-to-brain coupling and cognitive performance in response to these two different experimental conditions. This comparison should also allow to comprehend the significance of the positive feedback *per se*, separated by the competitive/cooperative task effect.

## Author Contributions

MB designed the research, supervised the experiment, analyzed the data and wrote the text. MEV realized the experiment, analyzed the data and wrote the text.

## Conflict of Interest Statement

The authors declare that the research was conducted in the absence of any commercial or financial relationships that could be construed as a potential conflict of interest.
